# Snakes and Snake Poison

**Published:** 1883-06

**Authors:** 


### SNAKES AND SNAKE POISON.

When the constitutional symptoms of snake-poisoning set in, there is little to be done except to try to steer the patient through their course by administering light foods and stimulants when necessary, and by resorting to artificial respiration in a few suitable cases. The system of treating a victim to snake-poisoning as if he were suffering from narcotic poisoning and keeping him moving about is most injurious, and the opposite course should always be adopted. Dr. Wall has found no benefit accrue from the injection of ammonia or the permanganate of potash into the blood, nor does he speak encouragingly of the treatment, so often followed in India and elsewhere, of giving large quantities of alcohol to the extent of producing intoxication.

With regard to the prevention of the mortality from snake-bite, the Indian Government relies on offering rewards for killing poisonous snakes, and the occupation of snake-killer is no doubt much more lucrative than that of rat-catcher in this country. The natives, who go about their work barefooted and generally more than half naked, are often too much under the influence of the ancient superstition of snake-worship to make war on snakes with a prospect of exterminating them. Sir Joseph Fayrer tells us that they often object to killing cobras which may have taken up their quarters in their houses lest some misfortune may fall on their house or family. "Should fear, and perhaps the death of some inmate by accident. prove stronger than superstition, the cobra may be caught, tenderly handled, and deported to some field, where it is released and allowed to depart in peace." The mongoose is the natural enemy of the snake, and although it seems to be as tamable as the cat, its depredations on the poultry yard will always prevent it taking the place of the cat in the Indian's household. Doctor Wall mentions a fact relative to the mode of attack of the mongoose on poisonous snakes which we do not remember seeing stated before, and which places that animal very high in the scale of animal intelligence.

"One of the Indian snakes' chief enemies, the mongoose, has no fear whatever of the poison, as any one will confess who has seen with what complete ease he seizes the snake and crushes out first one and then the other of the poison fangs with his long incisors, and then devours the cobra—thus rendered helpless—at his leisure. That the mongoose is perfectly aware of the existence of the poison fang there can be no doubt, for he only seizes the cobra by the fang; and should he miss his aim he retires at once out of reach to make a fresh attack."

Dr. Wall makes this statement in support of his theory that the poison fangs of snakes have not been developed for defensive purposes, but to enable them to secure their prey with greater ease and certainty. The cobra lives chiefly on frogs, which he secures by paralyzing them, while the daboia, living on small but active mammals, disables them by throwing them into convulsions. It is probable that we have allowed the importance of the poisonous symptoms of snake-bites to overshadow our judgment in our inquiries into the uses of the poison fang. The gland in which the poison is secreted is analagous to the parotid gland in other animals, and its secretion has probably some similar use to the saliva in the economy of the snake's digestion, and its action, even as a poison, may be due to some essential principle which may act in a similar manner to ptyaline, pepsin, or pancreatin, and only in a secondary way becomes an instrument of offense or defense.

It would be an interesting and a useful subject of inquiry for those persons who have the opportunity of making it whether snakes have, as the ancients asserted, strong antipathies to certain substances. Aristotle tells us that serpents may be driven away from a house by the smell of rue. Pliny says that the root of the holm-oak is an enemy to scorpions and that of the ash to serpents, which, however, will not return under fern. Serpents may be driven away by the burning of hair or stag's-horn, or the sawdust of the cedar, or a few drops of galbanum, green ivy or juniper, and those who are rubbed with juniper seeds are perfectly secure from hurt by serpents. It is probable that such substances as carbolic acid would be found useful for driving snakes away, as two or three drops placed in the mouth of a snake instantly destroys it.—*Saturday Review.*

---

Never affect to be "plain" or "blunt;" these are synonyms of brutality and boorishness. Such persons are constantly inflicting wounds which time cannot heal; they lose the sympathy of their fellow men and soon find themselves, as it were, alone in the world.

Abundant hair is not a sign of bodily or mental strength, the story of Sampson having given rise to the notion that hairy men are strong physically, while the fact is that the Chinese, who are the most enduring of all races, are mostly bald, and as to the supposition that long and thick hair is a sign and token of intellectuality, all antiquity, all mad-houses and all common observation are against it. The easily-wheedled Esau was hairy. The mighty Cæsar was bald. Long-haired men are generally weak and fanatical, and men with scant hair are the philosophers and soldiers and statesmen of the world.—*London Lancet.*

